# Prediction of Follicular Thyroid Neoplasm and Malignancy of Follicular Thyroid Neoplasm Using Multiparametric MRI

**DOI:** 10.1007/s10278-024-01102-0

**Published:** 2024-06-05

**Authors:** Bin Song, Tingting Zheng, Hao Wang, Lang Tang, Xiaoli Xie, Qingyin Fu, Weiyan Liu, Pu-Yeh Wu, Mengsu Zeng

**Affiliations:** 1 Department of Radiology, Zhongshan Hospital, Shanghai Medical Imaging Institute, Fudan University, No180, Fenglin Road, Xuhui District, 200032 Shanghai, China; 2 Department of Radiology, Minhang Hospital, Fudan University, No 170, Xinsong Road, Minhang District, 201199 Shanghai, China; 3 Department of Ultrasound, Minhang Hospital, Fudan University, No 170, Xinsong Road, Minhang District, 201199 Shanghai, China; 4 Department of Pathology, Minhang Hospital, Fudan University, No 170, Xinsong Road, Minhang District, 201199 Shanghai, China; 5 Department of General Surgery, Minhang Hospital, Fudan University, No 170, Xinsong Road, Minhang District, 201199 Shanghai, China; 6 GE Healthcare, MR Research China, Beijing, China

**Keywords:** Thyroid, Follicular neoplasm, Multiparametric MRI, Nomogram, Preoperative assessment

## Abstract

The study aims to evaluate multiparametric magnetic resonance imaging (MRI) for differentiating Follicular thyroid neoplasm (FTN) from non-FTN and malignant FTN (MFTN) from benign FTN (BFTN). We retrospectively analyzed 702 postoperatively confirmed thyroid nodules, and divided them into training (*n* = 482) and validation (*n* = 220) cohorts. The 133 FTNs were further split into BFTN (*n* = 116) and MFTN (*n* = 17) groups. Employing univariate and multivariate logistic regression, we identified independent predictors of FTN and MFTN, and subsequently develop a nomogram for FTN and a risk score system (RSS) for MFTN prediction. We assessed performance of nomogram through its discrimination, calibration, and clinical utility. The diagnostic performance of the RSS for MFTN was further compared with the performance of the Thyroid Imaging Reporting and Data System (TIRADS). The nomogram, integrating independent predictors, demonstrated robust discrimination and calibration in differentiating FTN from non-FTN in both training cohort (AUC = 0.947, Hosmer-Lemeshow *P* = 0.698) and validation cohort (AUC = 0.927, Hosmer-Lemeshow *P* = 0.088). Key risk factors for differentiating MFTN from BFTN included tumor size, restricted diffusion, and cystic degeneration. The AUC of the RSS for MFTN prediction was 0.902 (95% CI 0.798–0.971), outperforming five TIRADS with a sensitivity of 73.3%, specificity of 95.1%, accuracy of 92.4%, and positive and negative predictive values of 68.8% and 96.1%, respectively, at the optimal cutoff. MRI-based models demonstrate excellent diagnostic performance for preoperative predicting of FTN and MFTN, potentially guiding clinicians in optimizing therapeutic decision-making.

**Supplementary Information:**

The online version contains supplementary material available at 10.1007/s10278-024-01102-0.

## Introduction

Follicular thyroid neoplasm (FTN), primarily driven by mutations in the Rat sarcoma (RAS) gene family, encompasses a range of thyroid tumors– follicular thyroid adenoma (FTA), follicular tumors with uncertain malignant potential (FT-UMP), and follicular thyroid carcinoma (FTC) [1, 2]. Malignant follicular thyroid neoplasm (MFTN) often leads to a worse prognosis, such as local recurrence, distant metastases, and reduced overall survival [3, 4]. Thus, accurately diagnosing malignancy of FTN is crucial for informed clinical decision-making. For patients with thyroid nodules, ultrasonography serves as the primary method for assessing malignancy risk and guiding fine-needle aspiration (FNA) decisions. However, ultrasonography and biopsy frequently fall short in differentiating FTA from FTC [5]. Several studies have sought to identify ultrasonography features that could differentiate FTA and FTC, but results remain inconsistent [6, 7]. FNA, challenged by overlapping cytological features of FTC and FTA, often fails to provide a clear distinction [8, 9]. Therefore, there is a pressing need for an accurate, noninvasive, and preoperative prediction model for MFTN.

Distinguishing benign FTN (BFTN) from MFTN fundamentally requires differentiating FTN from non-FTN. Various Thyroid Imaging Reporting and Data System (TIRADS) based on ultrasonography risk factors, including the American College of Radiology TIRADS (ACR-TIRADS) [10], Korean TIRADS (K-TIRADS) [11], European TIRADS (EU-TIRADS) [12], Kwak-TIRADS [13], and Chinese-TIRADS (C-TIRADS) [14] are widely employed to manage thyroid nodules effectively. However, these systems are primarily established using malignant ultrasonography features of papillary thyroid carcinoma (PTC) rather than FTC [15, 16]. As the existing TIRADS classifications have shown ineffective in accurately identifying FTNs, Li et al. [17] proposed the need for a more effective risk stratification system. Lin et al. [16] highlighted the inefficiency of different TIRADS in managing patients with FTN, with 65.3–93.1% unnecessary biopsy rates, underscoring the need for FTN-specific stratification system. According to the American Thyroid Association guidelines, diagnostic surgical resection remains the standard treatment [18]; This approach predominately results in thyroidectomy for patients whose FNA cytology suggests a “follicular neoplasm” or “suspicious of follicular neoplasm”. However, it is notable that approximately 60–90% of these cases are benign [19, 20]. Hence, establishing an effective method for distinguishing FTN from non-FTN in thyroid nodules is imperative.

T2-weighted imaging (T2WI), dynamic contrast-enhanced magnetic resonance imaging (DCE-MRI), and diffusion-weighted imaging (DWI) anchor the core of multiparametric MRI [21]. Recently, the role of MRI has expanded, increasingly facilitating the preoperative evaluation of thyroid nodules and PTC [22–26]. MRI is recommended for evaluating thyroid nodules classified as TI-RADS category 4 or above, for cases with suspected aggressive thyroid cancer, and for large nodules where there is a need to distinguish between benign or malignant characteristics, evaluate cancer aggressiveness, and understand the nodule’s anatomical relationships with adjacent structures. However, there is a scarcity of research that focuses on multiparametric MRI features specific to patients with FTN. This study aims to evaluate the diagnostic efficacy of multiparametric MRI in distinguishing FTN from non-FTN and predicting FTN malignancy.

## Materials and Methods

### Patients and Study Design

The Institutional Review Board of our institute approved this study, and informed consent was exempted due to the study’s retrospective nature. We reviewed the medical records of consecutive patients who underwent thyroid nodule surgery from 2017 to 2022 in our institution. The inclusion criteria were the following: (1) enhanced thyroid MRI examination performed within one month before surgery; (2) complete pathology of postoperative specimens. The exclusion criteria were the following: (1) lesion < 5 mm; (2) poor image quality with motion or imaging artifacts that interfered with diagnosis; (3) missing images or postoperative pathological results; (4) bilateral diffuse lesions with different pathological types; (5) patients who underwent FNA or partial thyroidectomy before the MRI examination; (6) patients with unclear pathology; (7) patients with non-enhancing nodules considered a benign sign. Finally, this study included 702 thyroid nodules from 462 patients.

**Fig. 1:**
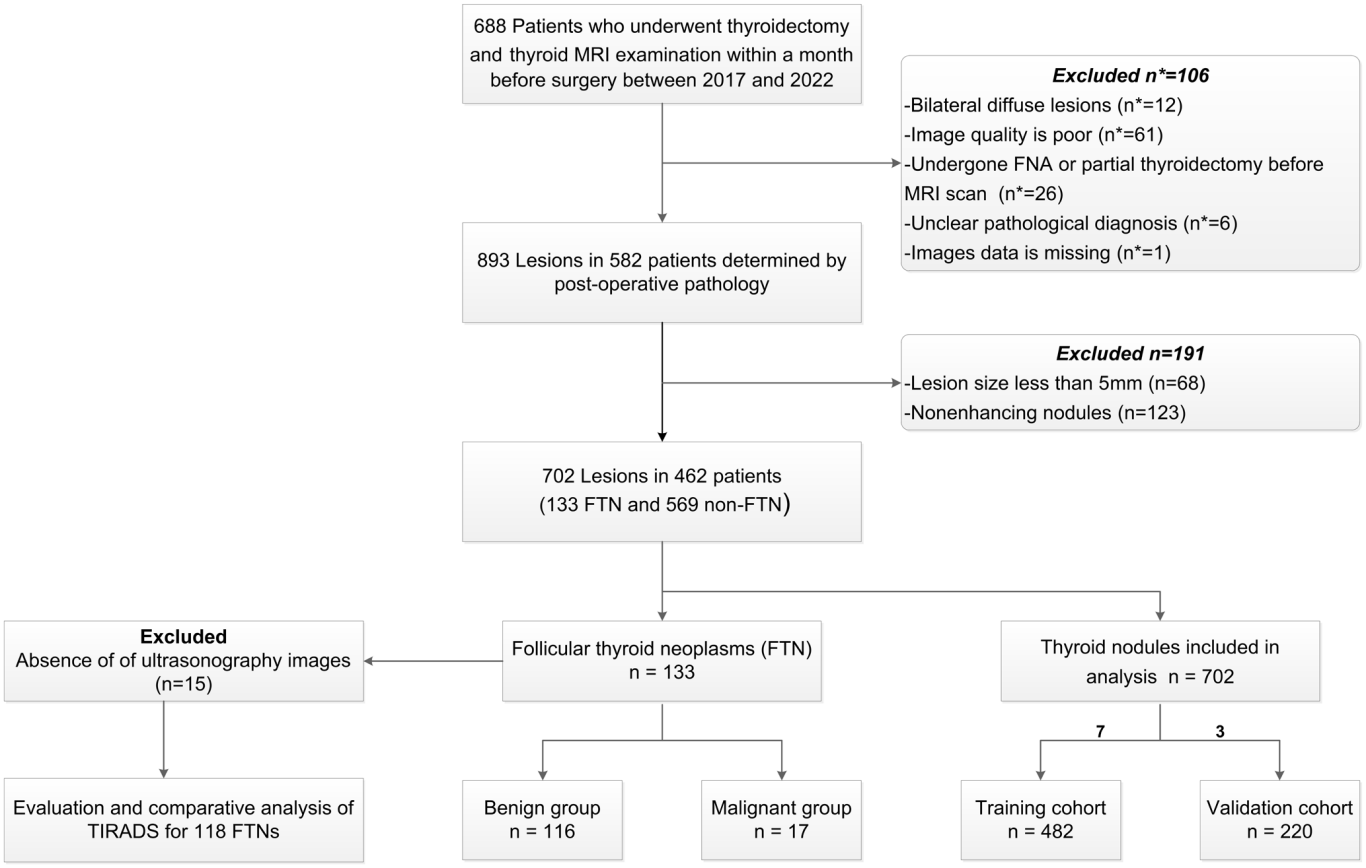
Study design and flowchart. *Abbreviations* n, number of lesions; n*, number of patients; MRI, Magnetic resonance imaging; FNA, fine needle aspiration; FTN, follicular thyroid neoplasm; non-FTN, non-follicular thyroid neoplasm

According to the postoperative pathology, lesions were split into FTN group (*n* = 133) and non-FTN group (*n* = 569). Seven hundred and two thyroid nodules were randomly divided into training cohort (482 thyroid nodules) and validation cohort (220 thyroid nodules) in a 7:3 ratio. In the same way, FTN was divided into an MFTN group (*n* = 17) and BFTN group (*n* = 116) according to postoperative pathology. BFTN included adenomatous goiter and FTA. MFTN included FT-UMP and FTC. Figure 1 shows the study flow diagram in detail.

### Magnetic Resonance Imaging Acquisition

MRI examinations were performed with a 1.5T MRI scanner from GE Healthcare (EXCITE HD) equipped with a customized 8-channel neck coil from Chenguang Medical Technology Ltd. The MRI sequences included (1) coronal and axial fat-saturated T2WI, (2) axial T1WI, (3) DWI (single-shot spin-echo echo-planar imaging) at b values of 0, 800–1000 s/mm², and (4) axial multiphasic contrast-enhanced T1WI. Gadolinium contrast medium (Magnevist; Bayer Healthcare, Berlin, Germany) was injected at 0.2 ml/kg and 3 ml/s, followed immediately by a 20-ml physiological saline flush. Images from six phases were acquired at 30 s, 60 s, 120 s, 180 s, 240 s, and 300 s after injection of the contrast agent, and the patients were instructed to hold their breath. Table 1 lists the detailed MRI acquisition parameters.

**Table 1:** Parameters of MRI Sequence

	Plane	TR	TE	Slice thickness	Gap between slices	NEX	FOV	Matrix size	Sequence
T2WI with fat suppression	coronal	1300	81.2	4	0.5	4	20	288 × 192	FRFSE
T1WI	axial	540	10.6	4	0.5	2	20	288 × 224	FSE-XL
T2WI with fat suppression	axial	3000	86.9	4	0.5	2	20	320 × 224	FRFSE
DWI	axial	6550	85	4	0.5	8	20	128 × 128	SE-EPI
Contrast-enhanced T1WI	axial	5.5	1.6	4	-2	1	25	256 × 192	FSPGR
Units		ms	ms	mm	mm		cm		

*Abbreviations* TR, repetition time; TE, echo time; NEX, number of excitations; FOV, field of view; T2WI, T2-weighted image; T1WI, T1-weighted image; DWI, diffusion-weighted imaging; FRFSE, fast recovery fast spin echo; FSE, fast spin echo; SS-EPI, single shot echo-planar imaging; FSPGR, fast spoiled gradient echo

### TIRADS

Two ultrasonography experts (with more than 10 years of experience) who were blinded to the histopathological results performed a retrospective analysis of ultrasonography features of thyroid nodules, and then they arrived at a consensus. The ultrasonography features included composition, echogenicity, margin, shape, echogenic foci, calcification, hypoechoic peripheral halo, extrathyroidal extension, and suspicious cervical lymph node. All thyroid nodules were categorized according to ACR-, K-, EU-, Kwak-, and C-TIRADS. For ACR-TIRADS, K-TIRADS and EU-TIRADS, category ≥ 4 or 5 was considered malignant; for Kwak-TIRADS and C-TIRADS, category ≥ 4b or 4c was considered malignant, respectively. The diagnostic performance of five TIRADS was calculated. Because of a lack of ultrasonography images, 15 FTNs were excluded. Finally, 118 FTNs were assessed based on five different TIRADS.

### Magnetic Resonance Imaging Analysis

Two radiologists with five and nine years of thyroid MRI experience independently interpreted the MRI findings using Advantage Workstation 4.5 (GE Healthcare, Waukesha, WI, USA) and picture archiving and communication system (PACS). To ensure a blinded study, the tumor histopathology results were kept from the MRI observers. The final decision was made by consensus if there was disagreement between two radiologists. Tumor size and number (unifocal or multifocal) were assessed (the largest linear dimension of nodules < 4 cm or ≥ 4 cm). The following qualitative MRI features were evaluated: (1) high signal intensity on T2WI, low signal intensity on T2WI, and high signal intensity on T1WI; (2) cystic degeneration; (3) flow-void signal; (4) restricted diffusion; (5) pseudocapsule; (6) reversed halo sign; (7) fissure-filling enhancement; (8) hyperintense on T2WI with enhancement; (9) uniformity of contrast enhancement (homogeneous or heterogeneous); (10) wash-out pattern; 11) hyperenhancement in early phase; 12) change of lesion in multiphasic enhancement. MRI feature definitions and diagrams are detailed in Supplementary materials.

### Model Establishment

First, we developed a nomogram for the prediction of FTN in the training cohort. The imaging factors underwent univariate analysis to determine the predictive factors related to the FTN. Subsequently, multivariate logistic regression was utilized to enhance the accuracy and reliability of FTN prediction. We selected the optimal model by Akaike Information Criteria. Models were developed based on the multivariate analysis.

Subsequently, a risk score system (RSS) for distinguishing BFTN and MFTN was constructed. The risk score was generated by utilizing the β coefficient of the logistic regression models for all factors that attained statistical significance in the multivariate analysis. To simplify the calculation process, the decimal was omitted, and the integer part of the β coefficient was taken. The sensitivity, specificity, accuracy, positive predictive value (PPV), and negative predictive value (NPV) of the RSS of MFTN at optimal cutoff value were compared with the same values from the TIRADSs. Using the net reclassification index (NRI), we evaluated the ability of different models to reclassify patients into the MFTN or BFTN group.

### Statistical Analysis

All statistical analyses were performed with SPSS Statistics 26.0 (IBM Corp, Armonk, NY, USA) and R software 4.2.0 (http://www.r-project.org). Continuous variables were represented as mean ± standard deviation (SD) or categorical variables as percentages. We compared continuous variables with an independent t-test. Categorical variables were compared by a Chi-square test or Fisher’s exact test. The Cohen’s Kappa test was used to assess the concordance between the two radiologists. By maximizing Youden’s index, the optimal cutoff value of the RSS for predicting MFTN was determined from a receiver operating characteristic (ROC) curve analysis. Bootstrap validation with 1000 resamples was adopted for evaluation of the RSS. Nomogram was constructed by the R software package “rms”. Hosmer-Lemeshow test was used to assess the model’s goodness-of-fit, with *P* ≥ 0.05 indicating a good fit. We conducted ROCs analyses, calibration curve analyses, and decision curve analyses (DCAs) to evaluate the performance of the nomogram. Statistical tests were performed with two-tailed p values, and P value < 0.05 was deemed statistically significant.

## Results

### Clinicopathological Characteristics

The mean patient age was 49.61 years (range: 12–84), and 133/702 (18.9%) were FTN and 569/702 (88.1%) were non-FTN. Table 2 lists the clinicopathological characteristics and MRI features of thyroid nodules in the training and validation cohort. There were no differences in clinicopathological features between the training cohort and the validation cohort except for lesion number (*P* = 0.026). In addition, among the 133 FTN patients with a mean age of 50.33 years (age range: 19–84), there were 116/133 (87.2%) BFTNs and 17/133 (12.8%) MFTNs. The BFTN group included 69/133 (51.9%) adenomatous goiter and 47/133 (35.3%) FTA. The MFTN group included 12/133 (9%) FTC and 5/133 (3.8%) FT-UMP.

**Table 2:** Comparisons of clinicopathologic characteristics and MR imaging features of patients in the training and validation cohort

Characteristic	Total	Training cohort	Validation cohort	P value
	(*n* = 702)	(*n* = 482)	(*n* = 220)	
Age, mean ± SD, years	49.61 ± 14.00	49.20 ± 14.12	50.51 ± 13.73	0.248
Gender				0.393
Male	174(24.8)	124(25.7)	50(22.7)	
Female	528(75.2)	358(75.3)	170(77.3)	
Hashimoto’s thyroiditis				0.553
Absent	580(82.6)	401(83.2)	179(81.4)	
Present	122(17.4)	81(16.8)	41(18.6)	
Lesion number				0.026
unifocal	236(33.6)	175(36.3)	61(27.7)	
multifocal	466(66.4)	307(63.7)	159(72.3)	
Tumor size				0.742
<4 cm	620(88.3)	427(88.6)	193(87.7)	
≥4 cm	82(11.7)	55(11.4)	27(12.3)	
High signal intensity on T2WI				0.439
Absent	299(42.6)	210(43.6)	89(40.5)	
Present	403(57.4)	272(56.4)	131(59.5)	
Low signal intensity on T2WI				0.124
Absent	453(64.5)	302(62.7)	151(68.6)	
Present	249(35.5)	180(37.3)	69(31.4)	
High signal intensity on T1WI				0.596
Absent	551(78.5)	381(79.0)	170(77.3)	
Present	151(21.5)	101(21.0)	50(22.7)	
Cystic degeneration				0.181
Absent	617(87.9)	429(89.0)	188(85.5)	
Present	85(12.1)	53(11.0)	32(14.5)	
Flow-void signal				0.455
Absent	661(94.2)	456(94.6)	205(93.2)	
Present	41(5.8)	26(5.4)	15(6.8)	
Restricted diffusion				0.507
Absent	418(59.5)	283(58.7)	135(61.4)	
Present	284(40.5)	199(41.3)	85(38.6)	
Pseudocapsule				0.505
Absent	456(65.0)	317(65.8)	139(63.2)	
Present	246(35.0)	165(34,2)	81(36.8)	
Reversed halo sign				0.968
Absent	430(61.3)	295(61.2)	135(61.4)	
Present	272(38.7)	187(38.8)	85(38.6)	
Fissure-filling enhancement				0.083
Absent	634(90.3)	429(89.0)	205(93.2)	
Present	68(9.7)	53(11.0)	15(6.8)	
Hyperintense on T2WI with enhancement				0.392
Absent	453(64.5)	306(63.5)	147(66.8)	
Present	249(35.5)	176(36.5)	73(33.2)	
Uniformity of contrast enhancement				0.478
Homogeneous	109(15.5)	78(16.2)	31(14.1)	
Heterogeneous	593(84.5)	404(83.8)	189(85.9)	
Wash-out pattern				0.044
Absent	392(55.8)	256(53.1)	136(61.8)	
Present	310(44.2)	226(46.9)	84(38.2)	
Hyperenhancement in early phase				0.082
Absent	591(84.2)	398(82.6)	193(87.7)	
Present	111(15.8)	84(17.4)	27(12.3)	
Change of lesion in multiphasic enhancement				0.239
Absent	246(35.0)	162(33.6)	84(38.2)	
Present	456(65.0)	320(66.4)	136(61.8)	
Thyroid nodules				0.071
FTN	133(18.9)	100(20.7)	33(15.0)	
Non-FTN	569(81.1)	382(79.3)	187(85.0)	
Pathological pattern				0.091
Nodular goiter	242(34.5)	152(31.5)	90(40.9)	
Papillary thyroid carcinoma	291(41.5)	210(43.6)	81(36.8)	
Follicular thyroid adenoma	47(6.7)	34(7.1)	13(5.9)	
Follicular thyroid carcinoma	12(1.7)	11(2.3)	1(0.5)	
Adenomatous goiter	69(9.8)	51(10.6)	18(8.2)	
Nodular hashimoto thyroiditis	16(2.3)	9(1.9)	7(3.2)	
Subacute thyroiditis	11(1.6)	5(1.0)	6(2.7)	
Medullary thyroid carcinoma	5(0.7)	4(0.8)	1(0.5)	
Follicular tumour of uncertain malignant potential	5(0.7)	4(0.8)	1(0.5)	
Undifferentiated carcinoma	2(0.3)	1(0.2)	1(0.5)	
Other	2(0.3)	1(0.2)	1(0.5)	

The data are presented as number of patients with the percentage in parentheses. *Abbreviations* FTN, follicular thyroid neoplasm; non-FTN, non-follicular thyroid neoplasm; SD, standard deviation; T2WI, T2-weighted imaging; T1WI, T1-weighted imaging

### Independent Predictive Factors of FTN and MFTN

**Fig. 2:**
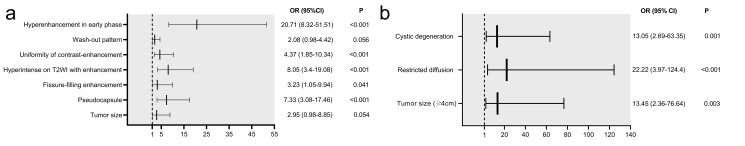
Forest plot of independent predictors for FTN **a** and MFTN **b** in multivariate logistic regression analysis

Table 3 shows factors associated with FTN in the training cohort and associated with MFTN on the basis of the FTN samples. The kappa values for interobserver agreement for all MRI features were from 0.725 to 1.000. The independent predictors of FTN were pseudocapsule (*P* < 0.001; OR = 7.33), fissure-filling enhancement (*P* = 0.041; OR = 3.23), hyperintense on T2WI with enhancement (*P* < 0.001; OR = 8.05), uniformity of contrast enhancement (*P* < 0.001; OR = 4.37), and hyperenhancement in early phase (*P* < 0.001; OR = 20.71). Tumor size (*P* = 0.003; OR = 13.45), restricted diffusion (*P* < 0.001; OR = 22.22), and cystic degeneration (*P* = 0.001; OR = 13.05) were independent predictors of MFTN in multivariate analysis. Figure 2 presents forest plots showing the multivariate analysis for FTN and MFTN.

**Table 3:** Univariate and multivariate analysis to identify factors associated with FTN in training cohort and MFTN in FTNs

Variables	Kappa value	FTN and non-FTN				BFTN and MFTN			
		Univariate analysis		Multivariate analysis		Univariate analysis		Multivariate analysis	
		Odds ratio (95% CI)	P value	Odds ratio (95% CI)	P value	Odds ratio (95% CI)	P value	Odds ratio (95% CI)	P value
Age (years)	1.000	1.00 (0.99–1.02)	0.509			0.98 (0.95–1.02)	0.274		
Gender (male, female)	1.000	1.09 (0.66–1.79)	0.743			2.93 (1.03–8.33)	0.044 *		
Hashimoto’s thyroiditis	1.000	0.84 (0.46–1.55)	0.588			1.44 (0.37–5.62)	0.597		
Lesion number	1.000	1.07 (0.68–1.7)	0.760			0.62 (0.22–1.71)	0.353		
Tumor size (<4 cm, ≥4 cm)	0.939	14.62 (7.69–27.82)	<0.001 *	2.95 (0.98–8.85)	0.054	16.01 (3.48–73.72)	<0.001 *	13.45 (2.36–76.65)	0.003 *
High signal intensity on T2WI	0.879	38.25 (11.92-122.76)	<0.001 *			NA	0.990		
Low signal intensity on T2WI	0.725	0.01 (0-0.08)	<0.001 *			NA	0.993		
High signal intensity on T1WI	0.884	1.25 (0.74–2.11)	0.401			1.99 (0.67–5.89)	0.217		
Cystic degeneration	0.920	1.6 (0.84–3.04)	0.153			11.92 (3.77–37.75)	<0.001 *	13.05 (2.69–63.32)	0.001*
Flow-void signal	0.831	60.00 (13.88-259.42)	<0.001 *			7.41 (2.48–22.13)	<0.001 *		
Restricted diffusion	0.914	0.21 (0.12–0.37)	<0.001 *			8.48(2.72–26.45)	<0.001 *	22.22 (3.97-124.46)	<0.001 *
Pseudocapsule	0.899	4.18 (2.64–6.63)	<0.001 *	7.33 (3.08–17.46)	<0.001 *	2.46 (0.76–8.01)	0.134		
Reversed halo sign	0.818	0.03 (0.01–0.11)	<0.001 *			NA	0.992		
Fissure-filling enhancement	0.874	32.56 (15.07–70.35)	<0.001 *	3.23 (1.05–9.94)	0.041 *	2.1 (0.75–5.9)	0.161		
Hyperintense on T2WI with enhancement	0.893	24.50 (12.81–46.87)	<0.001 *	8.05 (3.4-19.08)	<0.001 *	NA	0.992		
Uniformity of contrast-enhancement	0.780	5.24 (3.12–8.81)	<0.001 *	4.37 (1.85–10.34)	<0.001 *	1.99 (0.61–6.48)	0.255		
Wash-out pattern	0.909	2.69 (1.7–4.27)	<0.001 *	2.08 (0.98–4.42)	0.056	4.75 (1.04–21.76)	0.045 *		
Hyperenhancement in early phase	0.900	48.61 (25.31–93.36)	<0.001 *	20.71 (8.32–51.51)	<0.001 *	2.65 (0.72–9.75)	0.143		
Change of lesion in multiphasic enhancement	0.743	0.29 (0.18–0.45)	<0.001 *			0.37 (0.11–1.19)	0.094		

*Abbreviations* FTN, follicular thyroid neoplasm; non-FTN, nonfollicular thyroid neoplasm; BFTN, benign follicular thyroid neoplasm; MFTN, malignant follicular thyroid neoplasm; CI, confidence interval. NA, not applicable. * *P*<0.05

### Development and Validation of the Nomogram for Predicting FTN

**Fig. 3:**
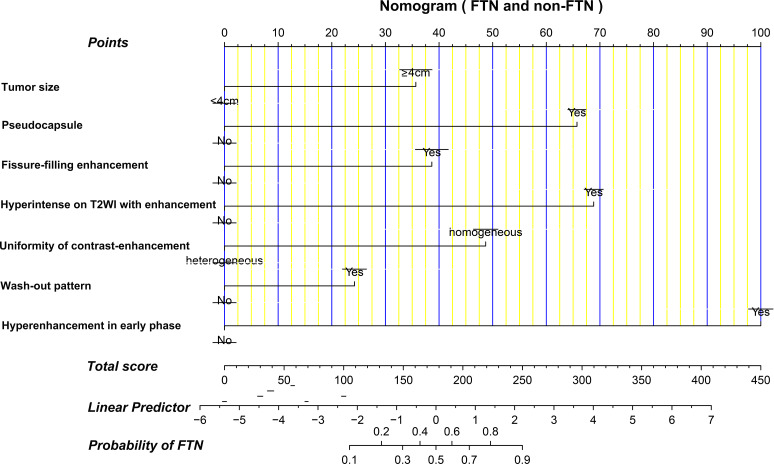
The nomogram for predicting the probability of FTN based on MRI features. When using the nomogram, draw a vertical line to the corresponding point on the axis for each variable. Then, a total score line displays the summed points for each variable. An individual probability of FTN is obtained by projecting the total score line onto the predicted probability bottom scale

**Fig. 4:**
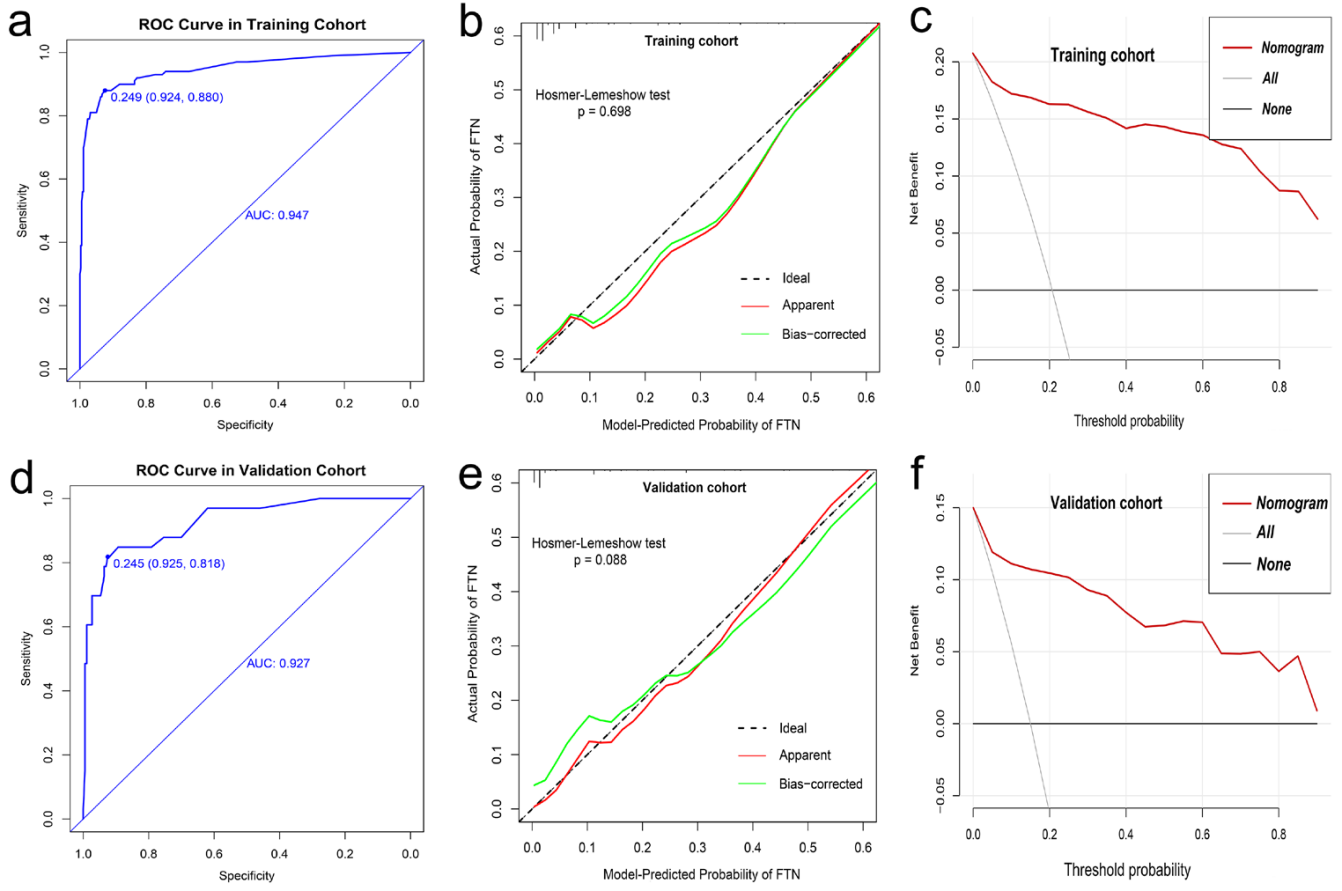
Performance and clinical utility of the nomogram for the discrimination of FTN and non-FTN. **a** ROC curve in training cohort (AUC: 0.947, 88% sensitivity and 92.4% specificity at the optimal cutoff value of 0.249); **b** Calibration curves in training cohort (Hosmer-Lemeshow test, *P* = 0.698); **c** Decision curves for the training cohort; **d** ROC curve in validation cohort (AUC: 0.927, 81.8% sensitivity and 92.5% specificity at the optimal cutoff value of 0.245); **e** Calibration curves in validation cohort (Hosmer-Lemeshow test, *P* = 0.088); **f** Decision curves for the validation cohort. ROC, receiver operating characteristic; AUC, area under the curve; FTN, follicular thyroid neoplasm

**Fig. 5:**
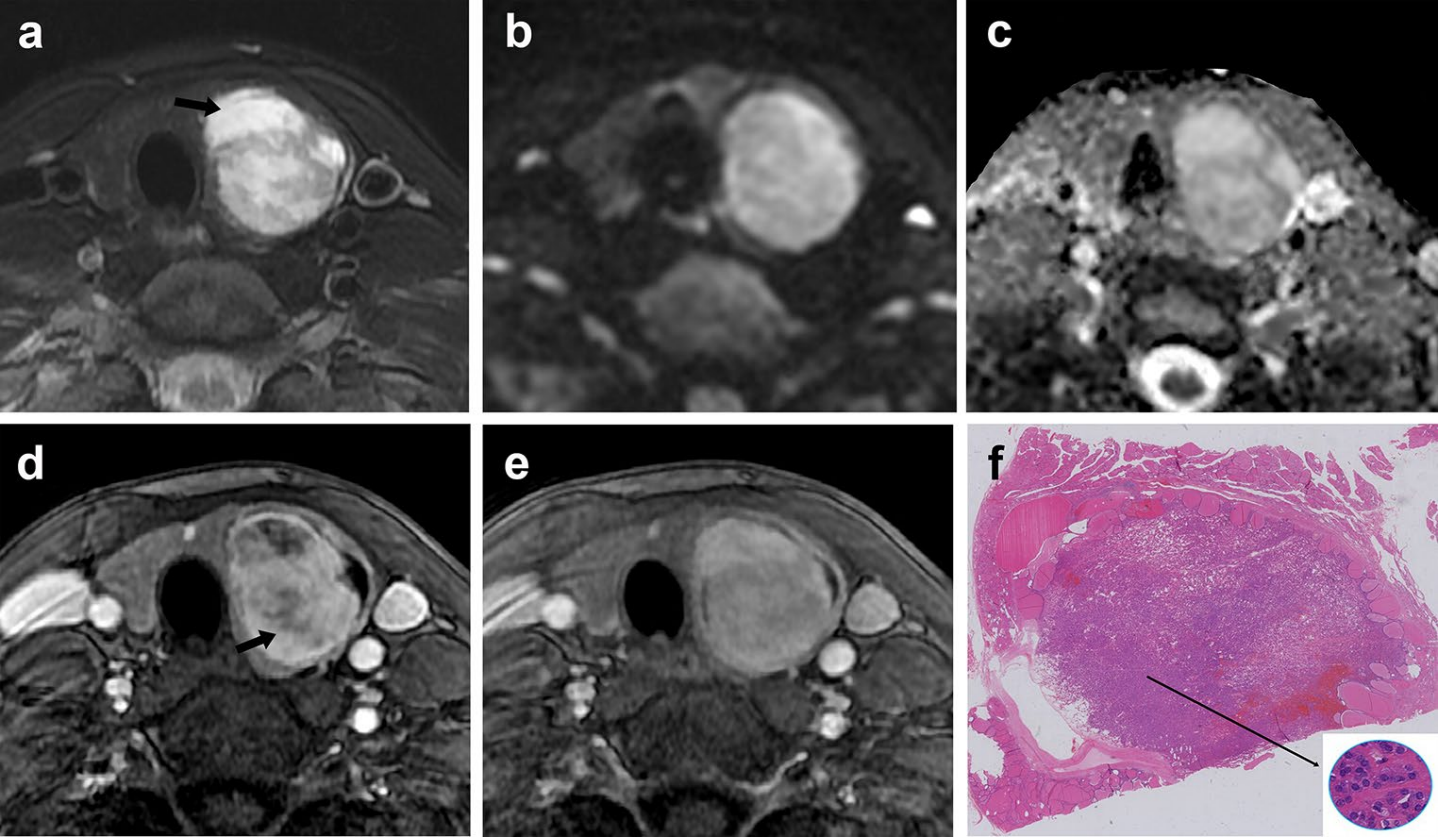
A 39-year-old woman presented with a thyroid follicular adenoma larger than 4 cm in the left lobe. Axial T2-weighted image **a** shows a heterogeneous mass and the present of hyperintense on T2WI with enhancement (black arrow). Axial DWI **b** and ADC **c** images show a hyperintense mass without restricted diffusion. Axial contrast-enhanced MRI (early phase) **d** shows a hyperintense enhancing mass and irregular fissure that did not enhance (black arrow). Axial contrast-enhanced MRI (delay phase) **e** shows a relatively homogeneous enhanced lesion with pseudocapsule, and fissure that do not enhanced in the early phase enhanced gradually. Pathological section analysis (HE, ×1) **f** shows densely distributed small follicles and relatively loose follicular cells (long black arrow, HE, ×40)

**Fig. 6:**
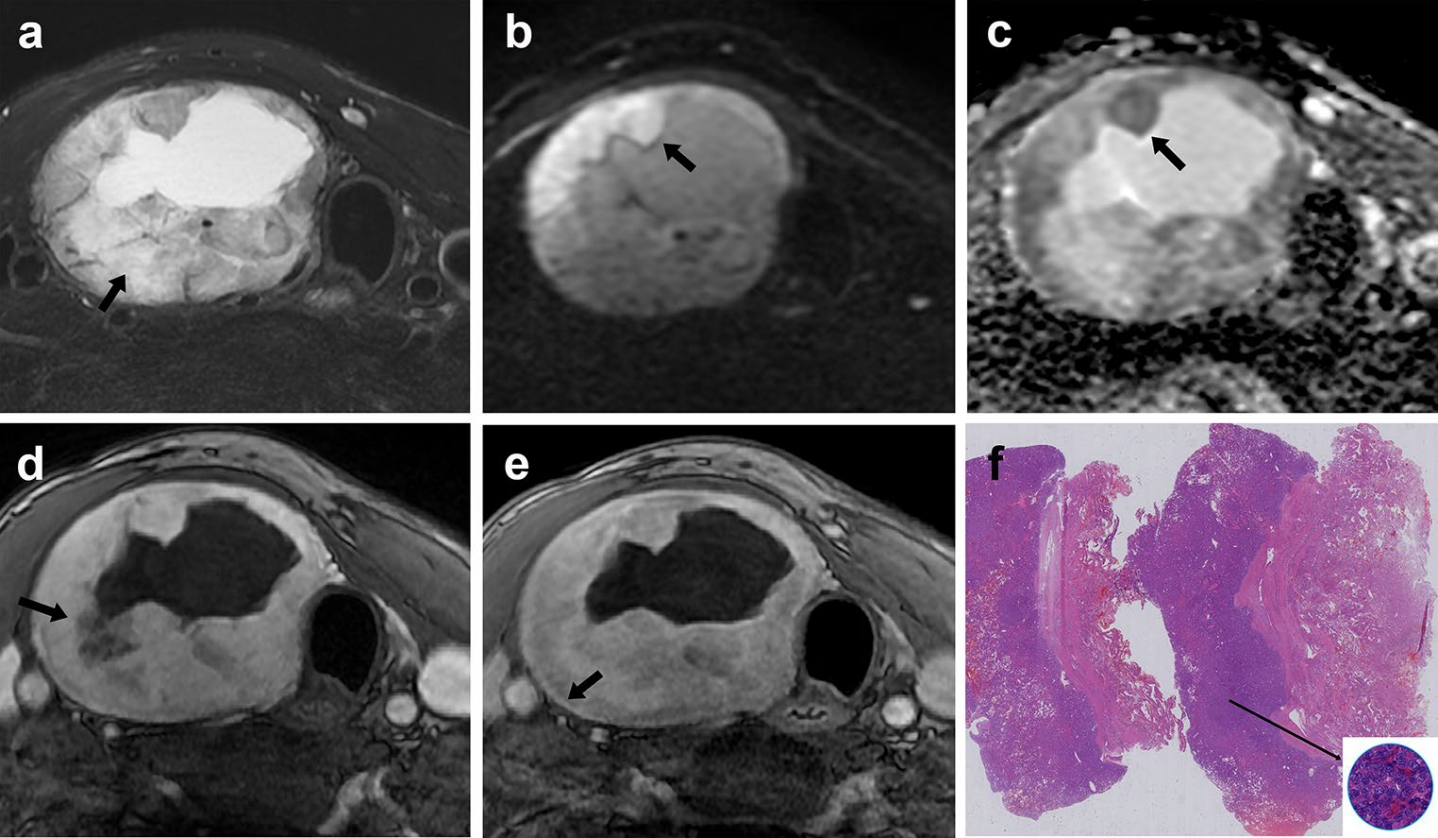
A 43-year-old man presented with a thyroid follicular carcinoma larger than 4 cm in the right lobe. Axial T2-weighted image **a** shows a heterogeneous mass and the present of hyperintense on T2WI with enhancement (black arrow). Axial DWI **b** and ADC **c** images show an area with restricted diffusion within the lesion (high signal on DWI and low signal on ADC, black arrow). Axial contrast-enhanced MRI (early phase) **d** shows a hyperintense enhancing mass and irregular fissure that did not enhance (black arrow). Axial contrast-enhanced MRI (delay phase) **e** shows a relatively homogeneous enhanced lesion with pseudocapsule (black arrow); fissure that do not enhanced in the early phase enhanced gradually, and the area of cystic degeneration is seen within the lesion. Pathological section analysis (HE, ×1) **f** shows densely distributed small follicles and follicular cells (long black arrow, HE, ×40)

Figure 3 shows the nomogram for predicting FTN and the corresponding areas under the curve (AUC) in the training and validation cohorts were 0.947 (95% CI: 0.919–0.976) and 0.927 (95% CI: 0.876–0.978), respectively (Fig. 4a and d). Figure 4b and e demonstrates the calibration plots of the nomogram for predicting FTN in the training and validation cohorts. The consistencies between the predicted and the actual probability of FTN are favorable. The nomogram for predicting FTN indicated a good fit with a P value of 0.698 in the training cohort and 0.088 in the validation cohort. The decision curves of the nomogram for predicting FTN in the training and validation cohorts (Fig. 4c and f) showed good clinical utility. Figures 5 and 6 illustrate representative MRI images of patients with FTN.

### Construction and Performance of the RSS for Predicting MFTN

**Fig. 7:**
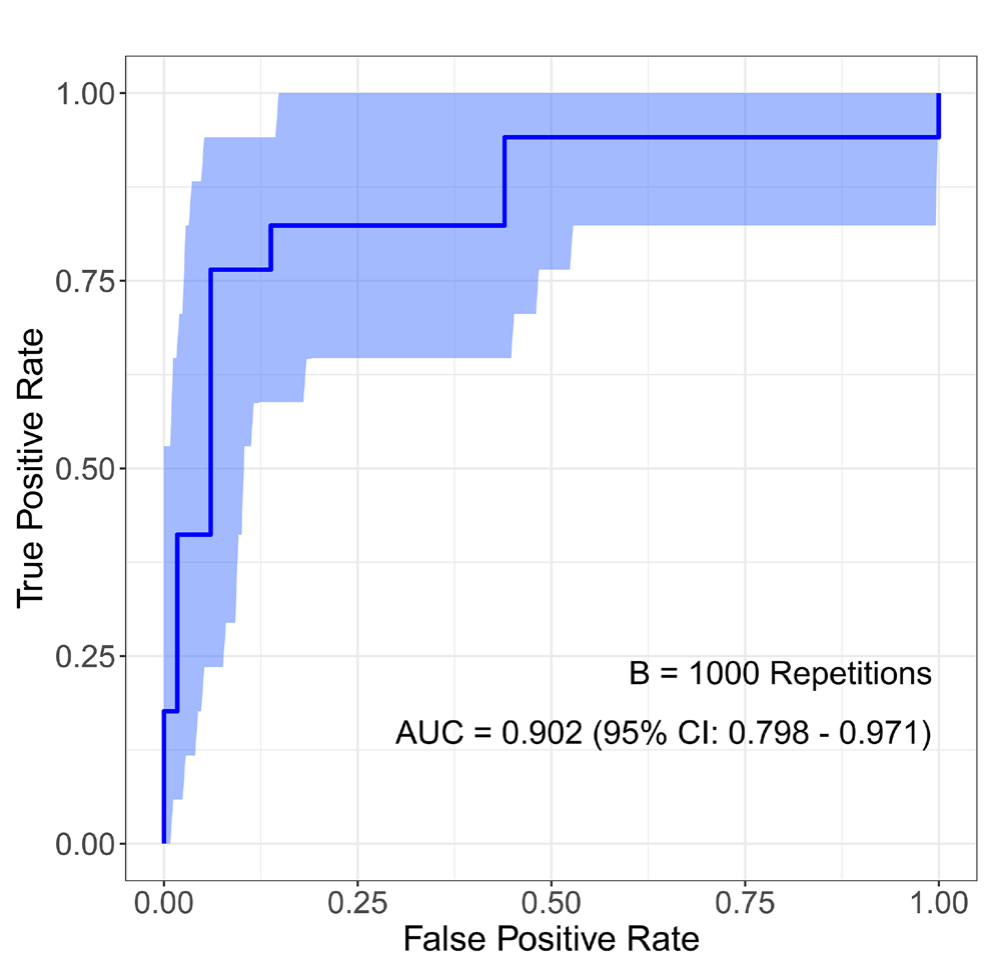
Internal validation of the risk score system (RSS) using the bootstrap sampling. The ROC curve was measured by bootstrapping for 1000 repetitions, and the AUC of the bootstrap stepwise model was showed

The RSS based on regression β coefficients from the preceding multivariate regression model was developed as follows: FTNs were given two points for tumor size, three points for restricted diffusion, and two points for cystic degeneration (Table 4). The risk score was calculated to stratify FTNs into high-risk group and low-risk group according to the optimal cutoff value determined by the ROC method. The ROC curve was measured by bootstrapping, and the bootstrap-corrected AUC was 0.902 (95% CI: 0.798–0.971) (Fig. 7). Table 5 presents diagnostic performance and ROC curves of the RSS and five different TIRADS for predicting MFTN in 118 FTNs. Sensitivity, specificity, accuracy, PPV, NPV, and AUC of the RSS at the optimal cutoff value of 3.5 were 73.3%, 95.1%, 92.4%, 68.8%, 96.1%, and 0.842, respectively. The diagnostic performance of the RSS exceeded the performance of several other ultrasonography-based TIRADS (AUC: 0.504–0.643). Compared with five TIRADS, the RSS showed a significant improvement for predicting MFTN based on NRI analysis (40.78 − 76.38%, *P* < 0.05).

**Table 4:** MRI-based risk score system to predict MFTN

Parameters	β coefficient	Score
Tumor size (<4 cm, ≥4 cm)	2.599	2
Restricted diffusion	3.101	3
Cystic degeneration	2.568	2

*Abbreviations* MFTN, malignant follicular thyroid neoplasm

**Table 5:** Diagnostic performance of RSS and five ultrasonography-based risk stratification systems to predict MFTN in 118 FTNs

Model (Cut-off value)	Sensitivity(%)	Specificity(%)	Accuracy(%)	PPV(%)	NPV(%)	AUC (95%CI)	NRI(%)	P
RSS (Cut-off = 3.5)	73.3	95.1	92.4	68.8	96.1	0.842 (0.706–0.979)		
ACR-TIRADS (≥ category 4)	73.3	55.3	57.6	19.3	93.4	0.643 (0.499–0.787)	40.78	0.041*
ACR-TIRADS (≥ category 5)	0	95.1	83.1	0	86.7	0.524 (0.373–0.676)	74.30	0.001*
K-TIRADS (≥ category 4)	46.7	57.3	55.9	13.7	88.1	0.520 (0.362–0.677)	65.50	0.003*
K-TIRADS (≥ category 5)	6.7	94.2	83.0	14.3	87.4	0.504 (0.346–0.662)	68.61	0.001*
EU-TIRADS (≥ category 4)	46.7	57.3	55.9	13.7	88.1	0.520 (0.362–0.677)	65.50	0.003*
EU-TIRADS (≥ category 5)	6.7	86.4	76.2	6.7	86.4	0.535 (0.385–0.685)	76.38	<0.001*
Kwak-TIRADS (≥ category 4b)	46.7	61.2	59.3	14.9	88.7	0.539 (0.381–0.697)	61.62	0.005*
Kwak-TIRADS (≥ category 4c)	0	94.2	82.4	0	86.6	0.529 (0.379–0.679)	75.28	0.001*
C-TIRADS (≥ category 4b)	26.7	82.5	75.4	18.2	88.5	0.546 (0.384–0.708)	60.26	0.008*
C-TIRADS (≥ category 4c)	0	97.1	84.7	0	87.0	0.515 (0.361–0.668)	72.36	0.001*

Data are presented as percent. PPV, positive predictive value; NPV, negative predictive value; AUC, area under the curve; NRI, Net reclassification index; FTN, follicular thyroid neoplasm; MFTN, malignant follicular thyroid neoplasm; RSS, risk score system; TIRADS, Thyroid Imaging Reporting and Data System; ACR, American College of Radiology. *P<0.05

## Discussion

In our study, we identified pseudocapsule, fissure-filling enhancement, hyperintense on T2WI with enhancement, uniformity of contrast enhancement, and hyperenhancement in early phase as key independent variables for FTN. The developed nomogram for FTN prediction demonstrated good discrimination performance, calibration, and clinical utility. For MFTN, tumor size, cystic degeneration, and restricted diffusion emerged as independent variables, with substantial interobserver agreement observed for these variables. Utilizing these aforesaid independent variables, we constructed RSS to predict MFTN. Comparative analysis using ROC and NRI indicated that the RSS outperformed the five main TIRADS classification systems in predicting MFTN.

The nomogram for FTN prediction exhibited impressive diagnosis performance in both training and validation cohorts. Current ultrasound-based malignancy risk stratification systems for thyroid nodules have proven inadequate in accurately characterizing FTNs, often resulting in unnecessary biopsies [16]. FTN patients might benefit from this nomogram by reducing the need for invasive biopsy. In our study, the presence of pseudocapsule emerged as a significant independent risk factor for FTN prediction, and appearing in 61% of FTNs in the training cohort, a higher prevalence compared to non-FTNs. The pseudocapsule is characterized as a fibrous connective tissue ring formed by tumor compression, which manifests as a halo sign on ultrasonography. Li et al. [27] reported that about 74% FTN had a pseudocapsule. We also found fissure-filling enhancement to be a distinctive feature, present in only 1.9% of non-FTNs but in about 42.9% of FTNs. Histologically, FTNs exhibit loose follicular structures, fibrous stroma, and hemorrhage internally, with fewer vessels, contrasting with densely distributed follicular structures, cells, and abundant vessels in the surrounding area. This contrast explains the pathological mechanism behind fissure-filling enhancement. Additionally, hyperintense on T2WI with enhancement and hyperenhancement in early phase were crucial in predicting FTN. The liquid-rich internal areas of FTNs contribute to hyperintense on T2WI with enhancement. The characteristic of dense area inside FTNs is that most of the cells are obviously crowded and/or accompanied by microfollicular formation [28]. These crowded cells contain proliferative-positive cells and, along with neovascularity, lead to hyperenhancement in early phase.

Differentiating MFTN from BFTN poses a significantly clinical, radiological, and histological challenge. The findings of our research indicated that the detection of MFTNs using the five ultrasound TIRADS stratification systems yielded a limited number of true positive cases, leading to reduced sensitivity and PPV. Supporting our observations, Lin et al.’s research into the efficacy of ultrasound-based malignancy risk stratification systems for thyroid nodules, specifically within FTN patients, revealed similar limitations. The sensitivity among K-TIRADS, EU-TIRADS, ACR-TIRADS, and C-TIRADS ranged from 26.9 to 38.8%, with the PPV ranging from 32.7 to 60% [16]. The AUC of five TIRADS stratification systems to predict MFTN ranged from 0.515 to 0.643 in our present study, which was comparable to findings of Lin et al. [16] who reported an AUC of 0.573 to 0.611. These results emphasized a broader challenge within the field in effectively diagnosing MFTNs through current TIRADS systems, thus highlighting the need for the urgent development of a malignant risk prediction model specially designed for FTNs. To address this, researchers have explored various preoperative methods to preoperatively distinguish FTC from FTA, such as serum-based analysis [29], radiomics and machine learning based on ultrasonography or CT images [5, 30, 31], and employing specific ultrasonography features [7, 27, 32, 33]. Nonetheless, the effectiveness of ultrasonography is constrained by physician experience, and its diagnostic performance is marked by notable interobserver variability [34]. In our study, we developed an RSS using multiparametric MRI features to differentiate MFTN from BFTN. The RSS exhibited a strong predictive capacity for MFTN, achieving an AUC value of 0.902.

Tumor size emerged as a crucial predictor for MFTN in our study. We categorized tumor size as a binary variable, setting the threshold at 4 cm. Aligning with Mu et al. [35], our results affirm tumor size as an independent predictor for MFTN corroborating previous research [36–39]. Additionally, we identified cystic degeneration as another independent predictor for MFTN. While cystic degeneration acts as a protective factor against malignancy in non-FTNs [40, 41], it poses a risk for malignancy in FTNs. Our findings are consistent with Ou et al. [33], whose results associated cystic degeneration with FTC. Contrasting results in other studies, particularly those focusing on ultrasonography features, might stem from varying definitions of cystic degeneration or differences in interobserver variability.

In oncology, diffusion-weighted imaging (DWI) is increasingly utilized for diagnosis, monitoring, and predicting outcome of malignancies [42]. DWI enables quantification of water diffusivity through the apparent diffusion coefficient (ADC), which is inversely correlated with cell density in tumor tissues [43]. ADC has become a valuable tool for distinguishing benign from malignant thyroid nodules [44]. However, despite the merits of the ADC, clinical practice seldom reports quantitative ADC values, and achieving consistent ADC measurements across different MRI scanners remains a challenge. We defined restricted diffusion as enhanced lesion areas appearing hyperintense on DWI and hypointense on ADC. Compared to quantitative ADC values, assessing restricted diffusion offers a practical and straightforward approach. In our study, restricted diffusion stood out as the most significant independent predictor. Pathologically, MFTN comprises of densely packed follicular cells, which limit the movement of water molecules.

Our study had several limitations. Firstly, as a single-center retrospective investigation, our results might be influenced by selection bias. Secondly, the small sample size of MFTN and the lack of analysis stratified by MFTN subtypes limit the conclusiveness of our findings; a larger MFTN sample is essential to validate the accuracy of the RSS for both benign and malignant FTN. Thirdly, while the MRI-based imaging features we identified are clinically practical, their subjectivity nature could have impacted our results. Lastly, reviewing static ultrasound images and reports may introduce bias, differing from real-life clinical practice.

## Conclusion

In conclusion, our nomogram, integrating MRI-based imaging features, excelled in accurately predicting FTN preoperatively, offering a potential reduction in invasive biopsies. Additionally, the RSS developed for MFTN prediction could significantly aid clinicians in refining therapeutic decision-making.

## Electronic Supplementary Material

Below is the link to the electronic supplementary material.

Supplementary Material 1

## Data Availability

The data sets generated and/or analyzed in the current study were not made public because patients’ personal information was included. Available from the corresponding author upon reasonable request.

## Abbreviations

MRI: Magnetic resonance imaging
US: Ultrasonography
FTN: Follicular thyroid neoplasm
non-FTN: Non-follicular thyroid neoplasm
MFTN: Malignant follicular thyroid neoplasm
BFTN: Benign follicular thyroid neoplasm
FTA: Follicular thyroid adenoma
FTC: Follicular thyroid carcinoma
PTC: Papillary thyroid carcinoma
FNA: Fine-needle aspiration
FT-UMP: Follicular tumors with uncertain malignant potential
TIRADS: Thyroid Imaging Reporting and Data System
ACR-TIRADS: American College of Radiology Thyroid Imaging Reporting and Data System
K-TIRADS: Korean Thyroid Imaging Reporting and Data System
EU-TIRADS: European Thyroid Imaging Reporting and Data System
C-TIRADS: Chinese Thyroid Imaging Reporting and Data System
ATA: American Thyroid Association
T1WI: T1 weighted imaging
T2WI: T2 weighted imaging
CI: Confidence intervals
SD: Standard deviation
ROC: Receiver operating characteristic
DCA: Decision curve analysis
DWI: Diffusion-weighted imaging
ADC: Apparent diffusion coefficient
PPV: Positive predictive value
NPV: Negative predictive value
NRI: Net reclassification index
