# Supplementary material for: Prediction of Follicular Thyroid Neoplasm and Malignancy of Follicular Thyroid Neoplasm Using Multiparametric MRI
Source: J Imaging Inform Med. 2024 Jun 5;37(6):2852–64. doi: 10.1007/s10278-024-01102-0 (PMC11612114; doi:10.1007/s10278-024-01102-0)
Supplement: Supplementary file 1 — Supplementary Material 1 [file 10278_2024_1102_MOESM1_ESM.docx]

**MRI features: definition and diagram**

1. **High signal intensity on T2WI**
   1. **The criteria for diagnosis:** high signal intensity on T2WI images can be seen in a focal or diffuse area of the thyroid nodule, similar to cerebrospinal fluid signal intensity.
   2. **The variable was indicated in the figures as follows:**

**
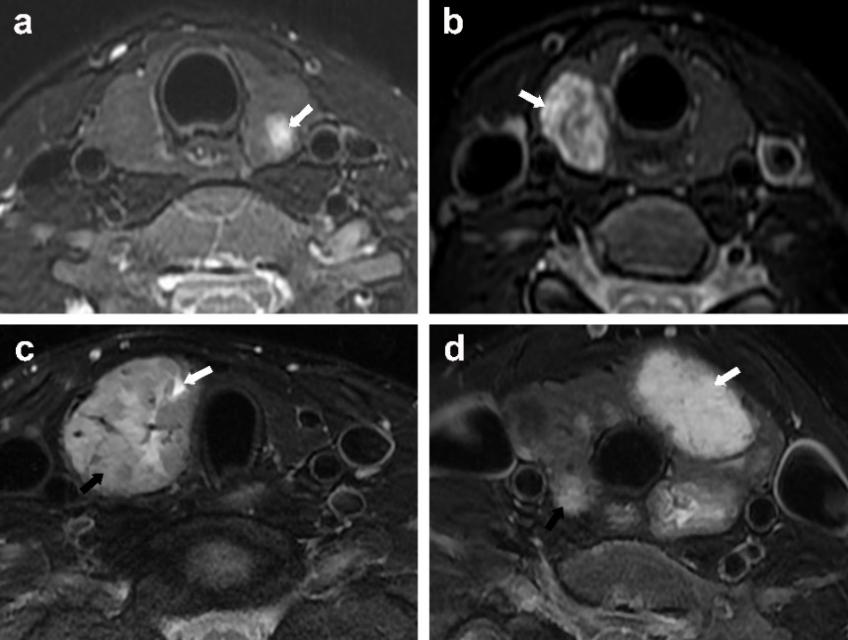
**

The white arrow refers to high signal intensity on T2WI from four different patients (a, b, c, d). **C**. the signal intensity (black arrow) is lower than that of cerebrospinal fluid, which does not meet the criteria of high signal intensity on T2WI.

1. **Low signal intensity on T2WI**
   1. **The criteria for diagnosis:** low signal intensity on T2WI images can be seen in a focal or diffuse area of the thyroid nodule.
   2. **The variable was indicated in the figures as follows:**

**
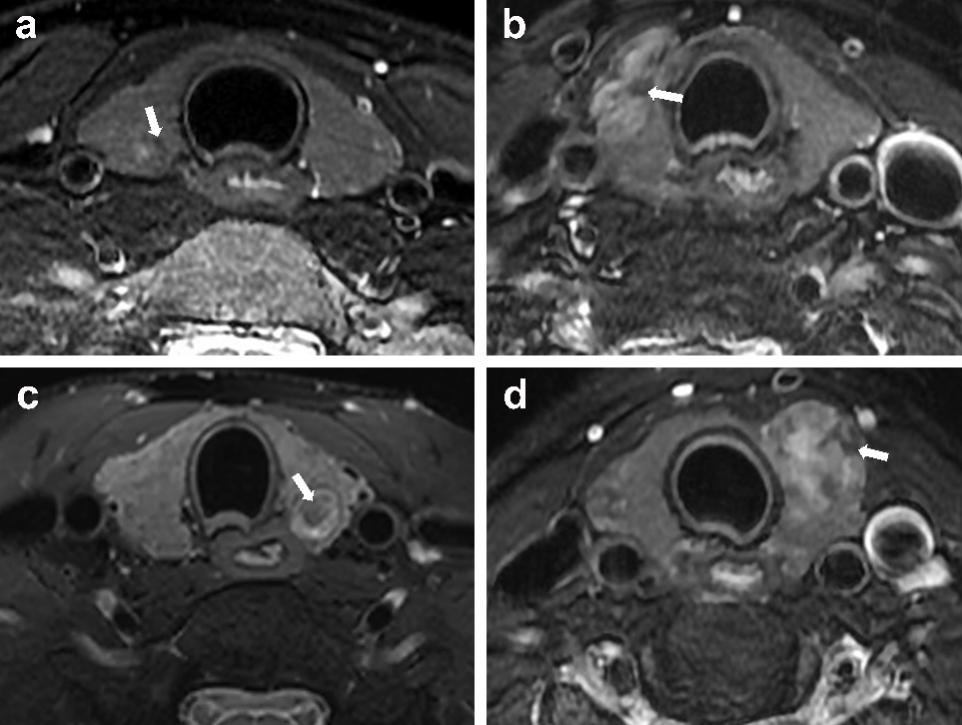
**

The white arrow refers to low signal intensity on T2WI from four different patients (a, b, c, d).

1. **High signal intensity on T1WI**
   1. **The criteria for diagnosis:** high signal intensity on T1WI images can be seen in a focal or diffuse area of the thyroid nodule.
   2. **The variable was indicated in the figures as follows:**

**
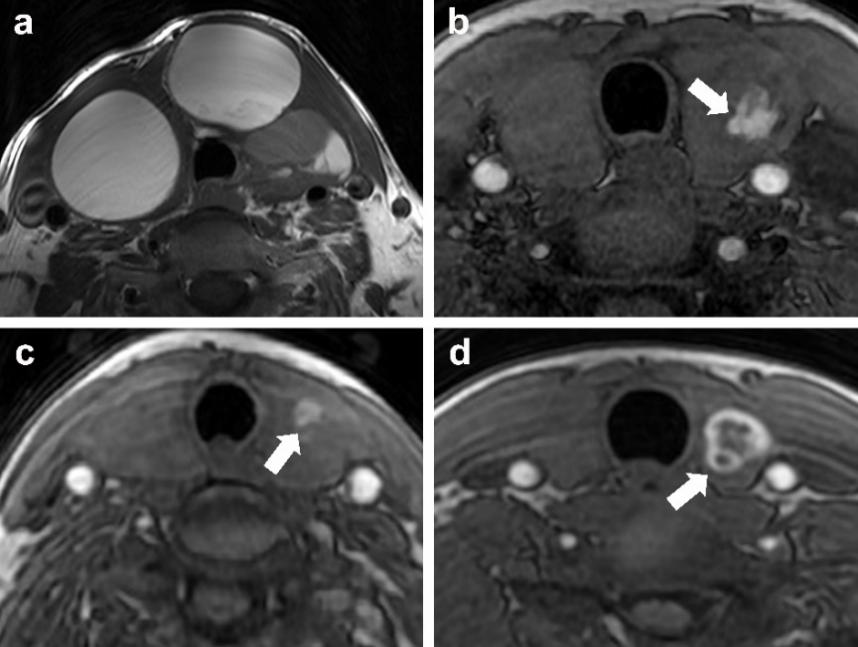
**

The white arrow refers to high signal intensity on T1WI from four different patients (a, b, c, d).

1. **Cystic degeneration** was identified as the area that was hypointense on pre-contrast T1-weighted images, markedly hyperintense on T2-weighted images and presented with non-enhancement, **which was indicated in the figures as follows:**


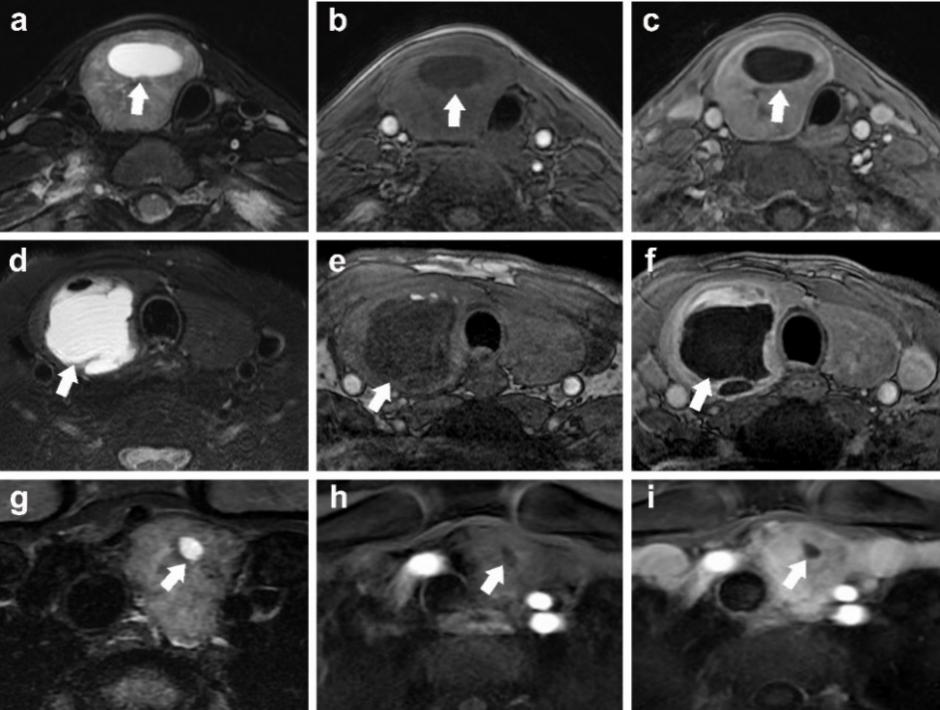


The images (a, b, c), (d, e, f) and (g, h, i) were obtained from the same patients and showed cystic degeneration (white arrows) that is markedly hyperintense on T2WI (a, d, g) and hypointense on T1WI (b, e, h); no enhancement was observed on the contrast-enhanced T1WI (c, f, i).

1. **Flow-void signal** was identified as multiple lines with no signal in the lesion on T2WI image, **which was indicated in the figures as follows:**

**
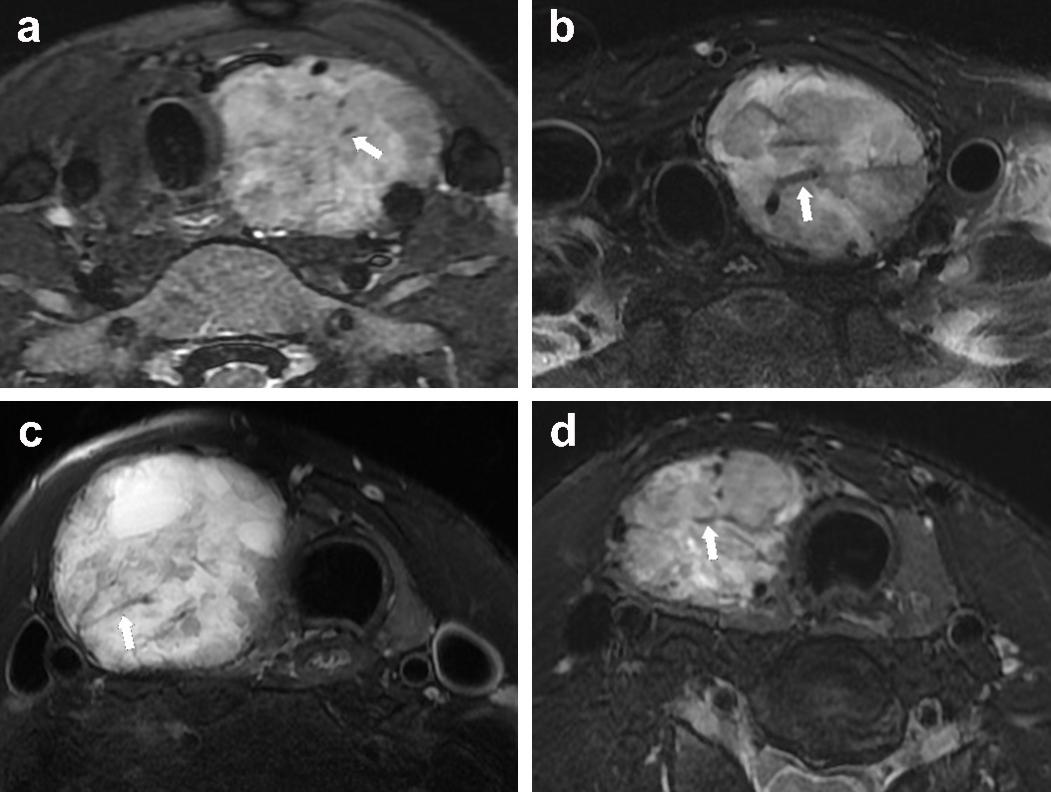
**

The white arrow refers to the flow-void signal from four different patients.

1. **Restricted diffusion** was identified as areas that were hyperintense or isointense on DWI images, hypointense on ADC maps, and presented with enhancement, **which was indicated in the figures as follows:**

**
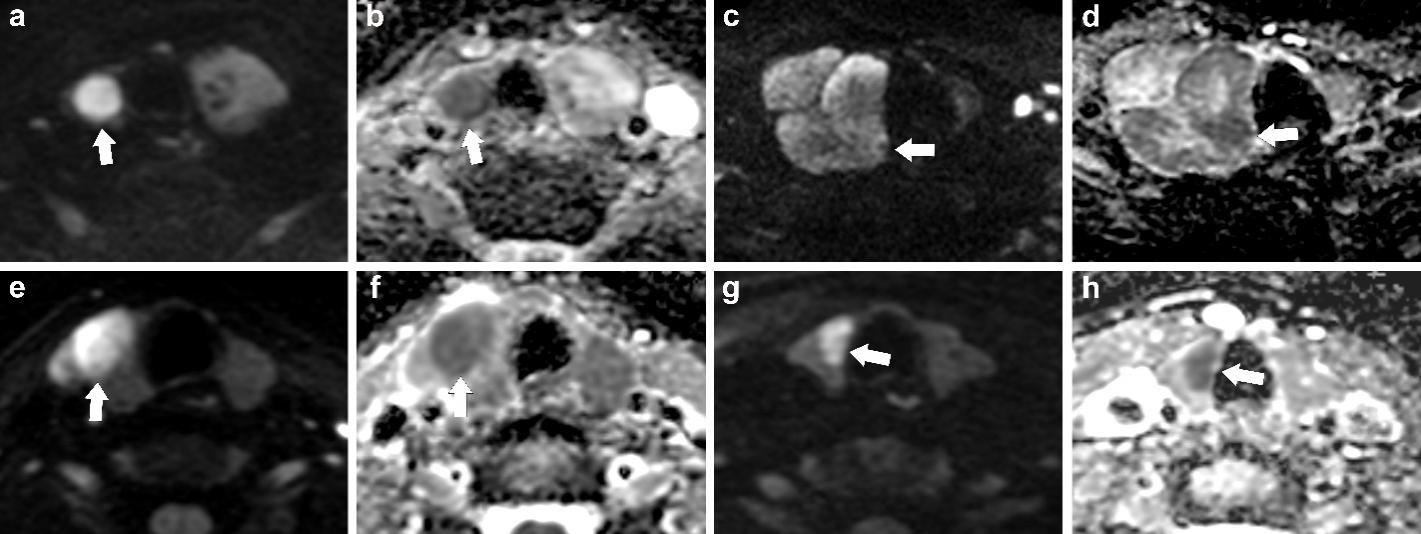
**

The images (a, b), (c, d), (e, f) and (g, h) were obtained from the same patients and showed restricted diffusion that is hyperintense on DWI (a, c, e, g) and hypointense on ADC (b, d, f, h) (white arrows).

1. **Pseudocapsule** was defined as a peritumoral thin rim that showed enhancement in multiphasic contrast-enhanced MRI imaging, **which was indicated in the figures as follows:**


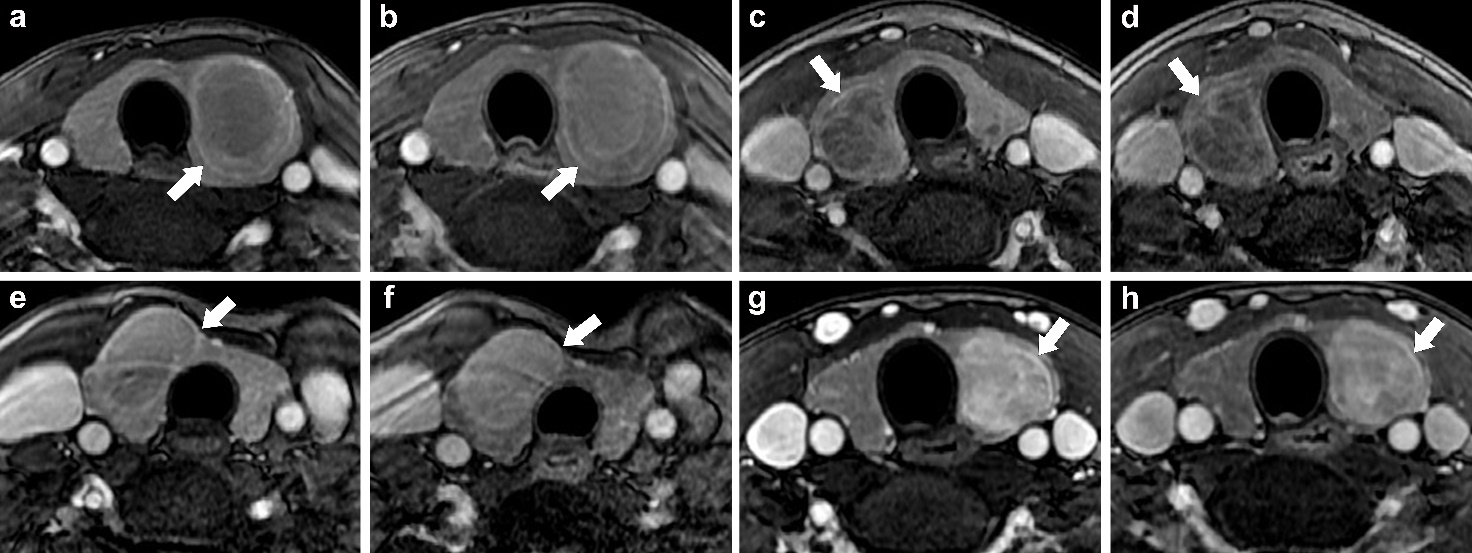


The early phase (a, c, e, g) and delay phase (b, d, f, h) of multiphasic contrast-enhanced MRI show pseudocapsule sign (white arrows), and the images (a, b), (c, d), (e, f) and (g, h) were obtained from the same patients.

1. **Reversed halo sign** was defined as the enhancement pattern in which the enhancement of the peripheral area of the lesion was greater than that of the central area in delay phase of multiphasic contrast-enhanced MRI, and the demarcation between areas was blurred, **which was indicated in the figures as follows:**

**
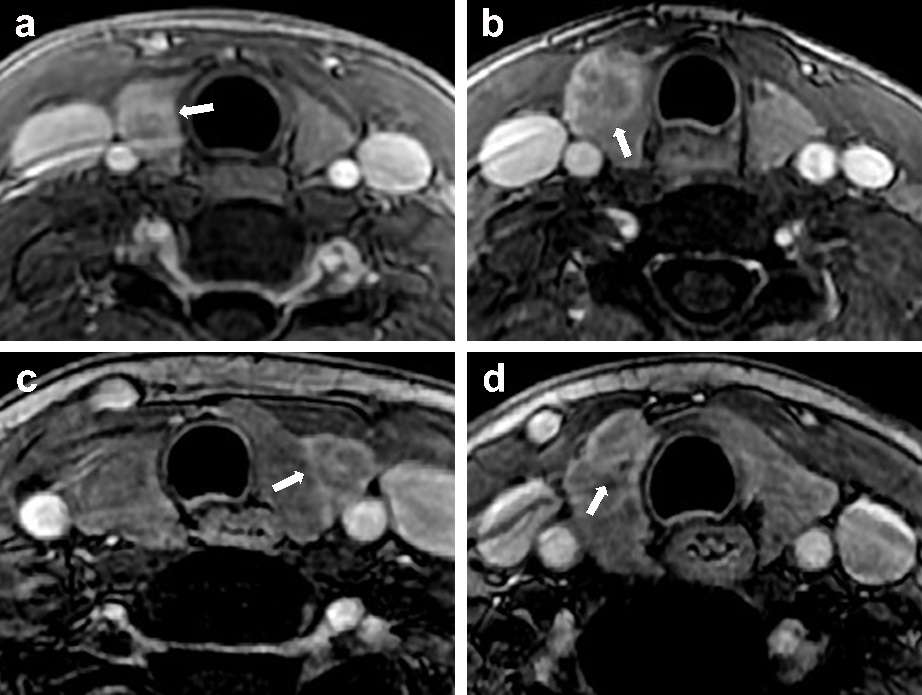
**

The white arrow refers to the reversed halo sign in delay phase from four different patients.

1. **Fissure-filling enhancement** was defined as the irregular fissure that did not enhance in the early phase in the internal area of the lesion but presented a progressive and filling enhancement pattern in the delay phase, **which was indicated in the figures as follows:**

**
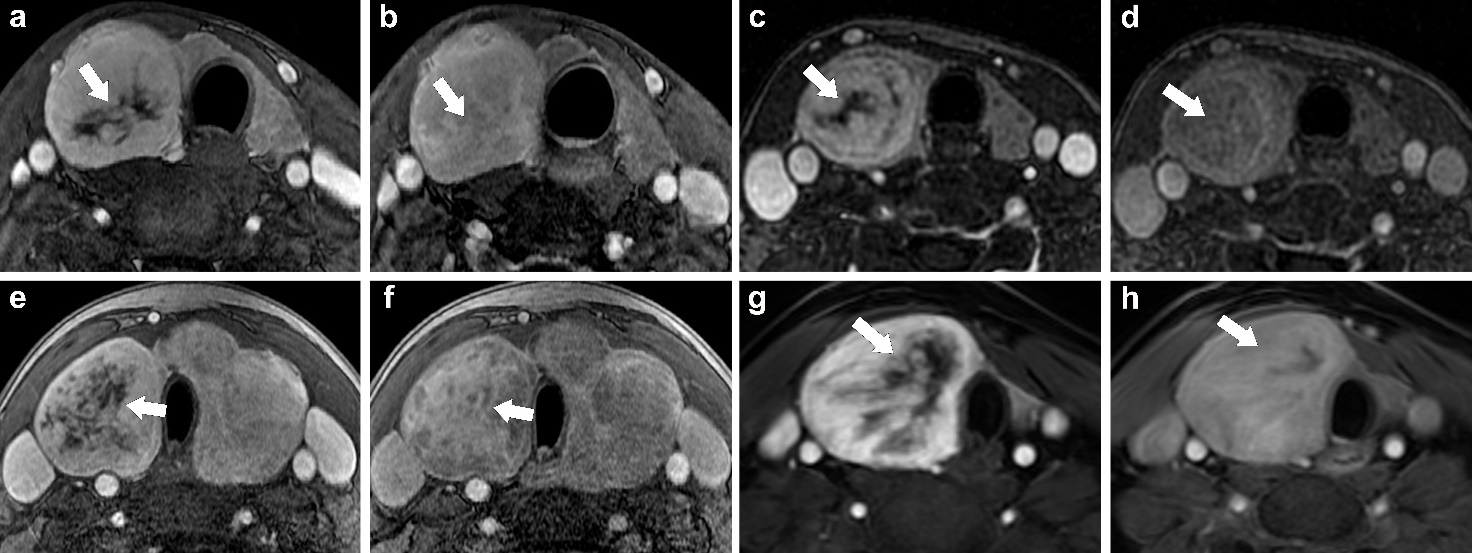
**

The early phase (a, c, e, g) and delay phase (b, d, f, h) of multiphasic contrast-enhanced MRI show fissure-filling enhancement (white arrows), and the images (a, b), (c, d), (e, f) and (g, h) were obtained from the same patients.

1. **Hyperintense on T2WI with enhancement** was defined as high signal similar to the cerebrospinal fluid signal on T2WI with enhancement in the enhanced MRI, **which was indicated in the figures as follows:**


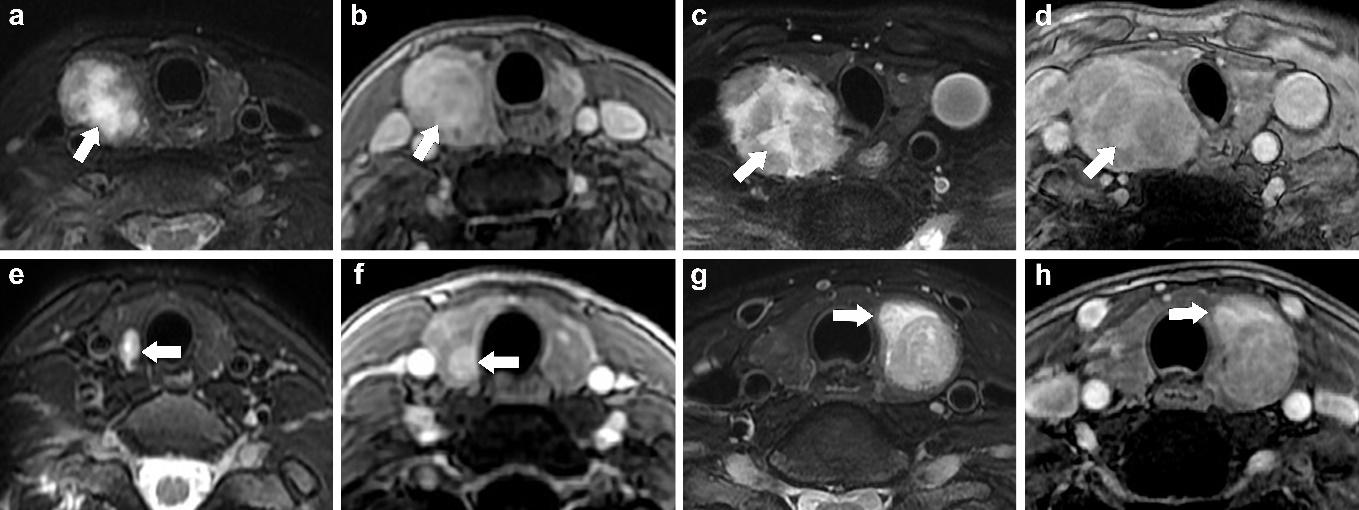


The images (a, b), (c, d), (e, f) and (g, h) were obtained from the same patients and showed hyperintense on T2WI with enhancement (white arrows) that is markedly hyperintense on T2WI (a, c, e, g) and enhancement was observed in the enhanced MRI (b, d, f, h).

1. **Uniformity** **of contrast-enhancement** was divided into homogeneous and heterogeneous, **which was indicated in the figures as follows:**


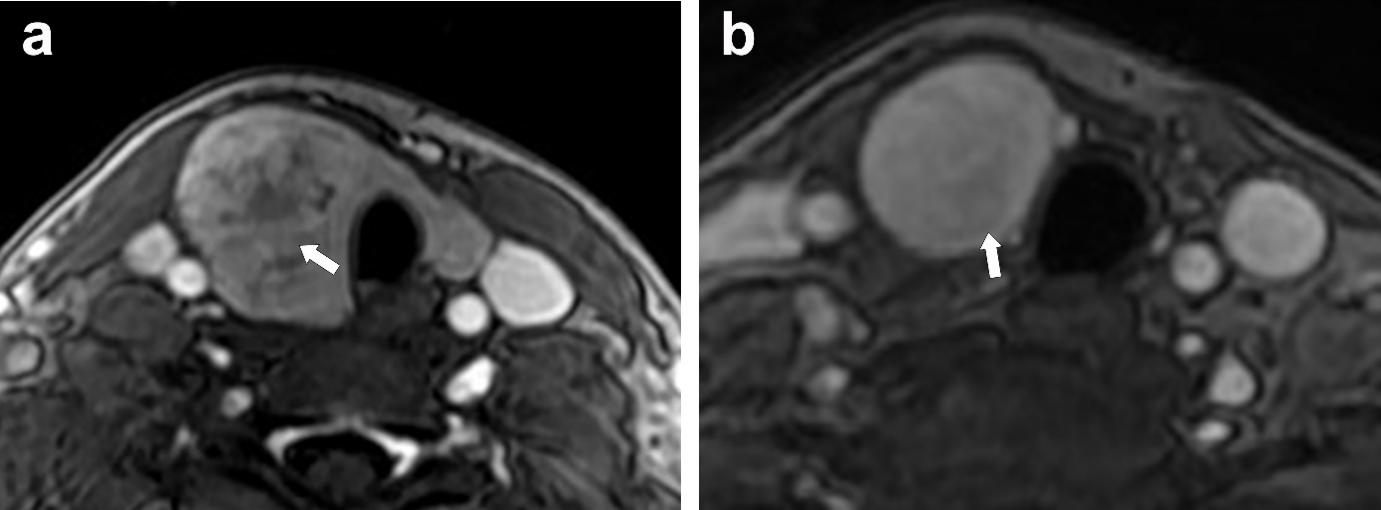


Contrast-enhanced T1WI showed heterogeneous (a) and homogeneous (b) enhancement (white arrows).

1. **Wash-out pattern** was defined as the enhancement of lesion in early phase of multiphasic contrast-enhanced MRI, and the degree of enhancement in delay phase decreased, **which was indicated in the figures as follows:**

**
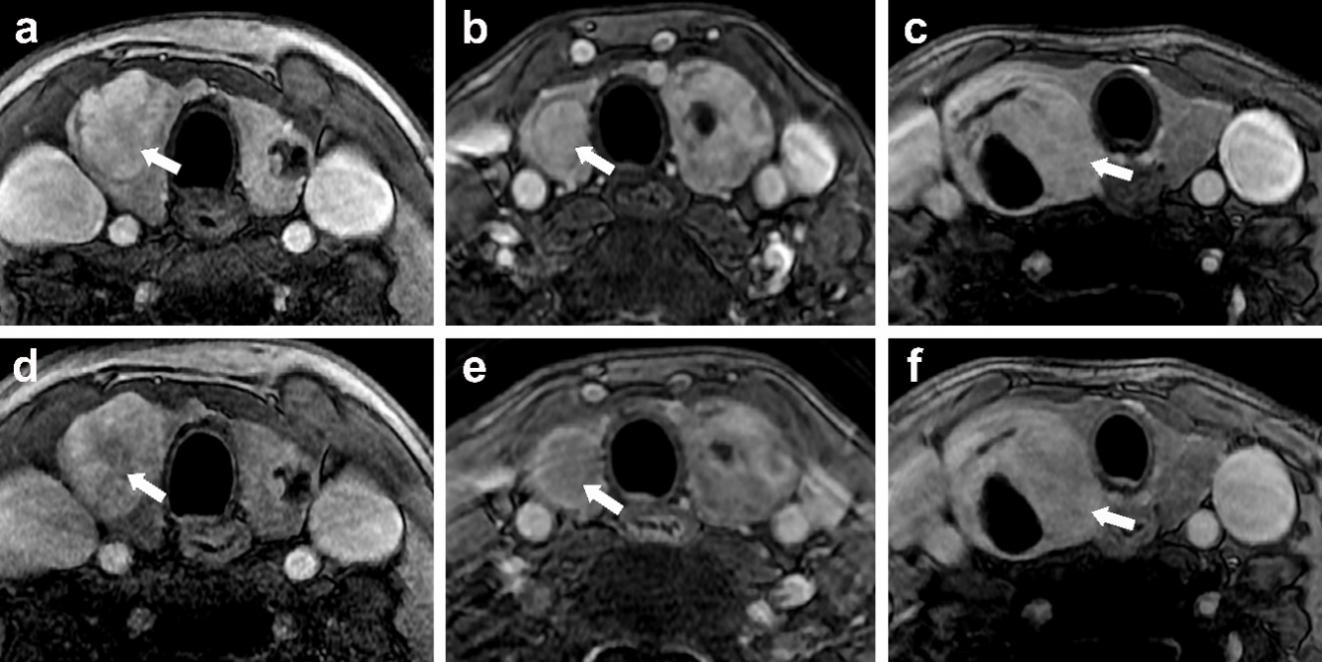
**

Wash-out pattern (white arrows) was observed in the early phase (a, b, c) and delay phase (d, e, f) of multiphasic contrast-enhanced MRI, and the images (a, d), (b, e) and (c, f) were obtained from the same patients.

1. **Hyperenhancement in early phase** was defined as the enhancement degree of lesion similar to that of the common carotid artery in the early phase of multiphasic contrast-enhanced MRI, **which was indicated in the figures as follows:**

**
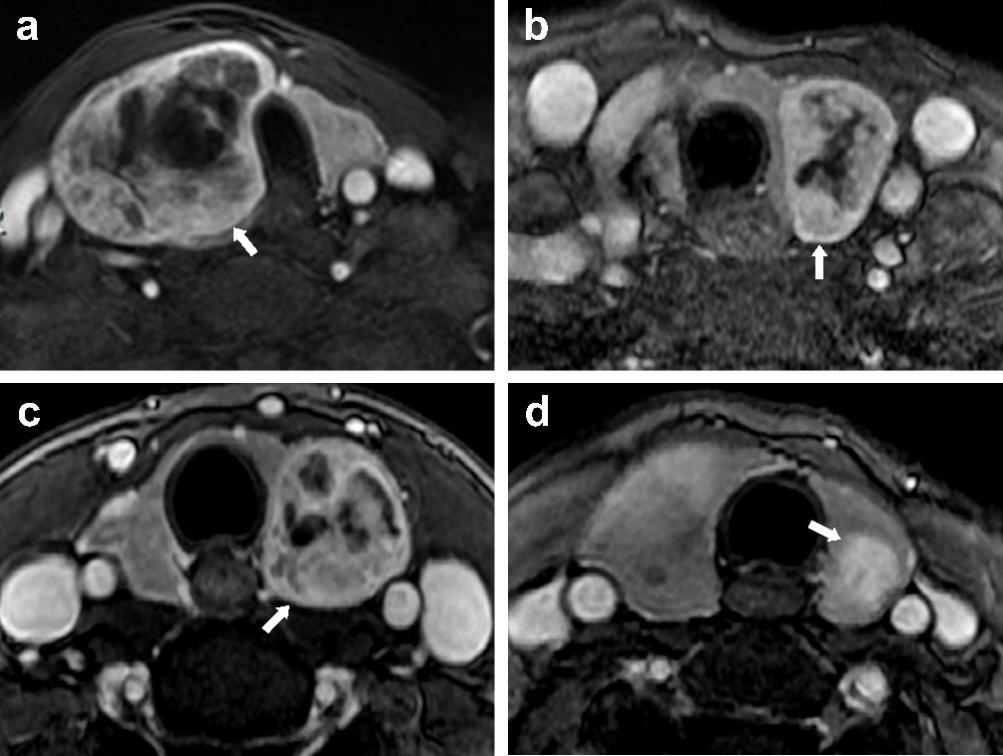
**

The white arrow refers to hyperenhancement in early phase from four different patients.

1. **Change of lesion in multiphasic enhancement** was defined as changes in the size and morphology of lesion in the early phase and delay phase of multiphasic contrast-enhanced MRI, **which was indicated in the figures as follows:**


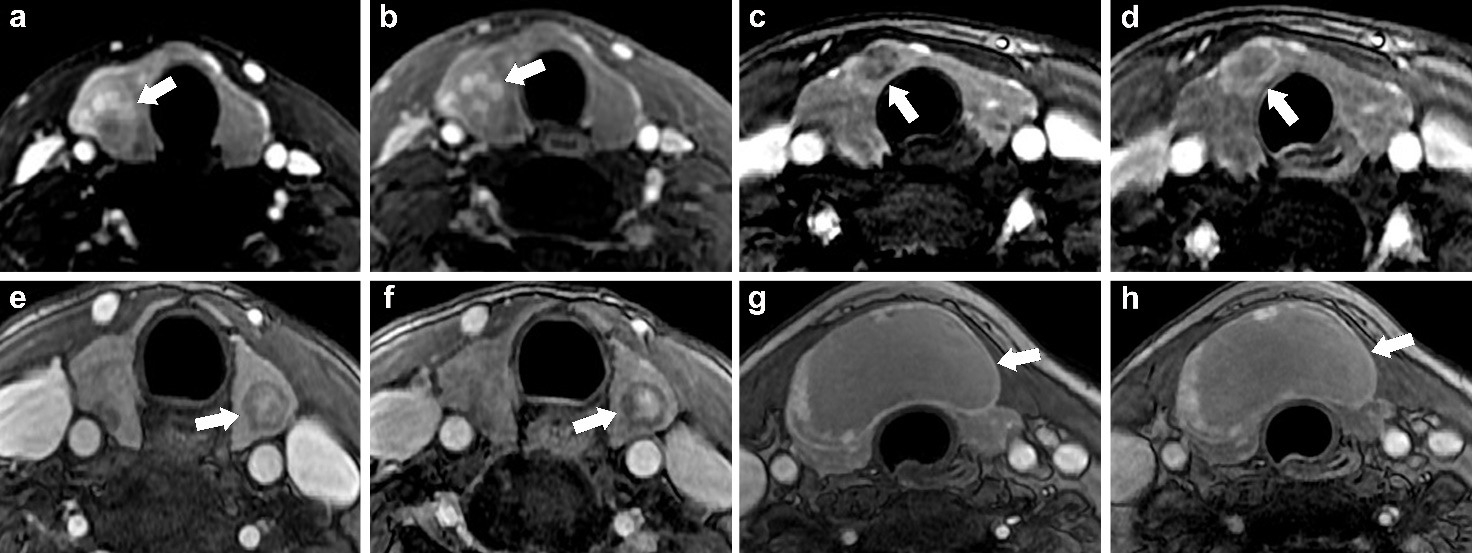


The images (a, b), (c, d), (e, f) and (g, h) were obtained from the same patients. The early phase (a, c, e, g) and delay phase (b, d, f, h) of multiphasic contrast-enhanced MRI showed change (a, b, c, d) and no change (e, f, g, h) of lesion size in multiphasic enhanced MRI.
